# Precision Diagnosis and Individualized Therapy of Non‐Gestational Choriocarcinoma Invading the Corpus Uteri and Cervix: A Case Report and Literature Review

**DOI:** 10.1002/cnr2.70393

**Published:** 2025-11-06

**Authors:** Jiahui Ma, Fenghua Ma, Tingting Chen, Xin Lu, Yan Du, Xiaoni Yue

**Affiliations:** ^1^ Clinical Research Unit Obstetrics and Gynecology Hospital of Fudan University Shanghai China; ^2^ Shanghai Key Lab of Reproduction and Development Shanghai China; ^3^ Shanghai Key Lab of Female Reproductive Endocrine Related Diseases Shanghai China; ^4^ Department of Radiology Obstetrics and Gynecology Hospital of Fudan University Shanghai China; ^5^ Department of Pathology Obstetrics and Gynecology Hospital of Fudan University Shanghai China; ^6^ Department of Gynecologic Oncology Obstetrics and Gynecology Hospital of Fudan University Shanghai China

**Keywords:** EMA‐CO, gestational choriocarcinoma, hCG, non‐gestational choriocarcinoma, short tandem repeat

## Abstract

**Background:**

Non‐gestational choriocarcinoma (NGCC) is a rare type of malignant tumor. Primary lesions are typically detected in the ovary and rarely invade the corpus uteri and cervix. NGCC usually has a poor prognosis due to the difficulty in achieving early and accurate diagnoses because of its rarity.

**Case:**

This case described a female who was initially diagnosed with gestational choriocarcinoma and treated with EMA‐CO chemotherapy. However, subsequent short tandem repeat (STR) analysis confirmed the diagnosis as NGCC, prompting surgical intervention. Given her favorable response and after thorough communication, three additional cycles of EMA‐CO chemotherapy were recommended. At her last follow‐up, her human chorionic gonadotropin level had normalized.

**Conclusion:**

This case presents a rare instance of NGCC with simultaneous uterine and cervical involvement, confirmed by STR analysis and successfully managed with the EMA‐CO regime. It highlights the necessity of precise diagnosis and personalized treatment for effective management of this disease.

## 1 Introduction

Choriocarcinoma is diagnostically established through clinical presentation and elevated serum human chorionic gonadotropin (hCG) levels. It is categorized into gestational (GCC) and non‐gestational choriocarcinoma (NGCC), depending on its pathogenesis. GCC represents the predominant form, with chemotherapy as the preferred treatment, often combined with adjuvant surgical intervention or radiotherapy [1]. NGCC, alternatively termed primary choriocarcinoma, is an extremely rare type of malignant tumor. While most commonly arising in the ovaries, extragenital manifestations typically occur along the body axis, particularly in the mediastinum or retroperitoneum. Current clinical practice adopts the International Federation of Gynecology and Obstetrics (FIGO) staging system based on the primary tumor site for therapeutic decision‐making [2].

Prognostically, GCC demonstrates excellent chemosensitivity when promptly treated, whereas NGCC carries a substantially poorer prognosis, largely attributable to diagnostic delays inherent to its rarity. Standard management of confirmed NGCC cases involves radical surgical resection combined with multi‐agent chemotherapy [2]. Notably, histopathological differentiation between GCC and NGCC remains clinically challenging, necessitating molecular confirmation through short tandem repeat (STR) analysis to guide appropriate therapeutic strategies.

This case report presents a case of NGCC with simultaneous involvement of both the uterine corpus and cervix, which was successfully treated with an individualized regimen at the Obstetrics and Gynecology Hospital of Fudan University, Shanghai, China. A complementary literature review was conducted to summarize current insights into the diagnosis and management of this disease.

## 2 Case

In January 2024, a 52‐year‐old postmenopausal woman (3 years since menopause, G3P1 with one spontaneous vaginal delivery in 1992 and two induced abortions in 1993 and 2014) was admitted to the Obstetrics and Gynecology Hospital of Fudan University complaining of a three‐month history of intermittent scanty vaginal bleeding. Initial transvaginal ultrasound at an outside hospital revealed a 61 × 62 × 45 mm cervical mass with 7 mm endometrial thickness.

**FIGURE 1:**
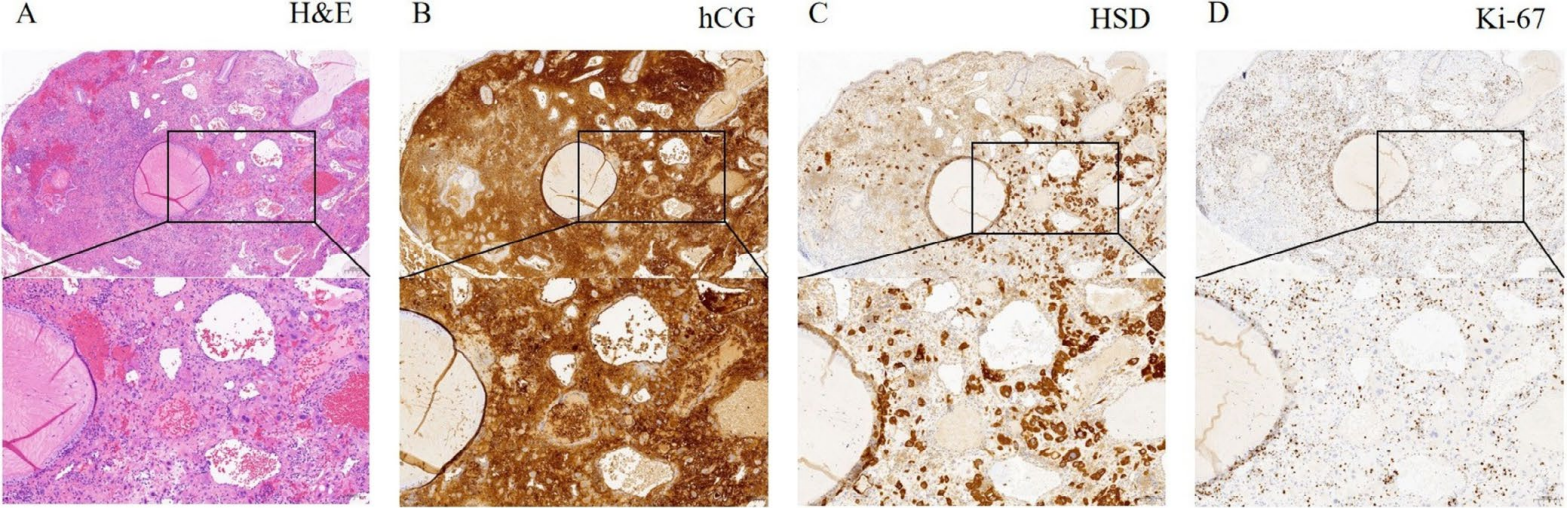
(A) Hematoxylin and Eosin staining was performed at magnifications of 4× and 10×. (B–D) Immunostaining of tumor cells showed positive expression of hCG, HSD, and Ki‐67, at the magnifications of 4× and 10×. hCG (+), HSD3B (+), Ki‐67 (+, 40%) supported the diagnosis of cervical choriocarcinoma.

**FIGURE 2:**
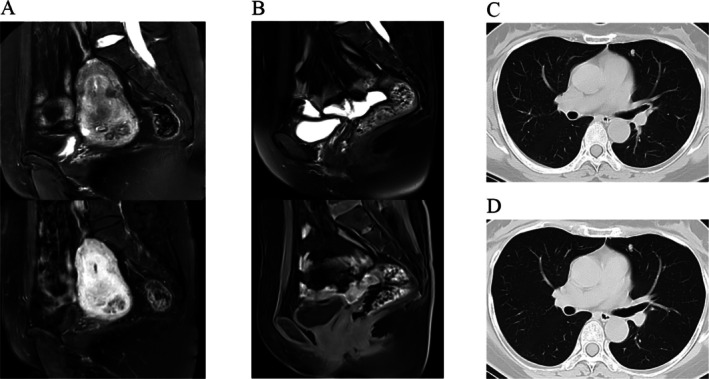
Imaging examination results before and after treatment. (A) Pelvic MRI revealed diffuse lesions within the uterine cavity and cervix, with the largest lesion measuring 7.6 cm in diameter. (B) The pelvic MRI demonstrated postoperative changes. (C) Chest CT revealed a solid nodule in the upper lobe of the left lung, with a maximum transverse diameter of 8 cm. (D) Chest CT again demonstrated that the size of the nodule was unchanged compared to earlier findings.

The patient's medical history was notable for HPV52 infection. Diagnostic workup included colposcopy, cervical biopsy, and endocervical curettage. Pathological examination suggested cervical choriocarcinoma, supported by immunohistochemical staining: hCG (+), HSD3B (+), Ki‐67 (+, 40%) (Figure 1A–D). Four days after the colposcopy, serum markers showed elevated hCG (15 366 mIU/mL) with normal SCCA (0.70 ng/mL) and CA125 (7.6 U/mL). Pelvic magnetic resonance imaging (MRI) revealed diffuse uterine and cervical lesions (maximum diameter: 7.6 cm) (Figure 2A), while chest computer tomography (CT) scan demonstrated an 8 × 5 mm lobulated nodule in the left upper lobe, suggestive of metastatic disease (Figure 2C). At diagnosis (III: 11), the serum hCG level had risen to 21 326 IU/L.

**FIGURE 3:**
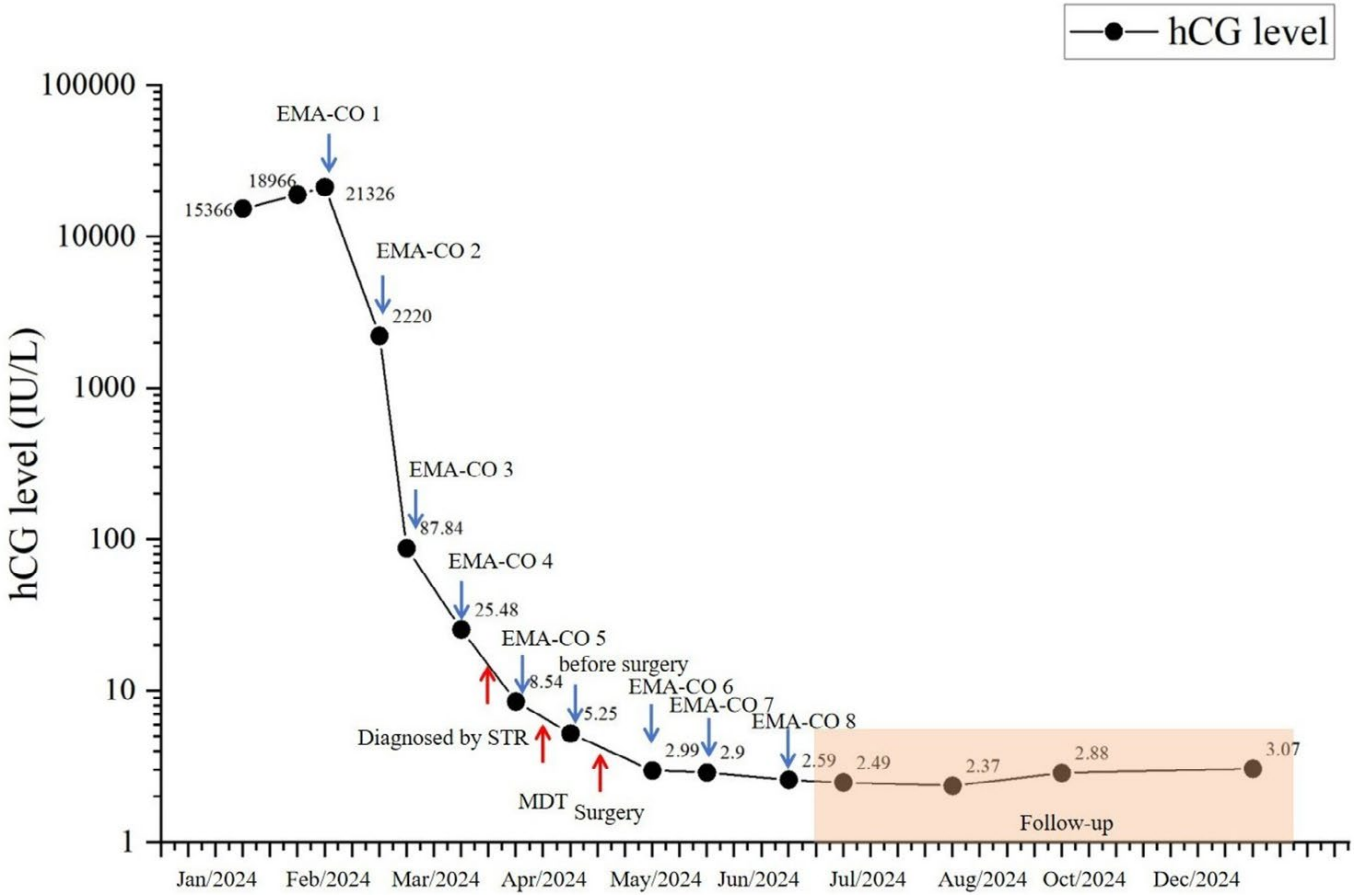
Changes of hCG levels throughout treatment.

**FIGURE 4:**
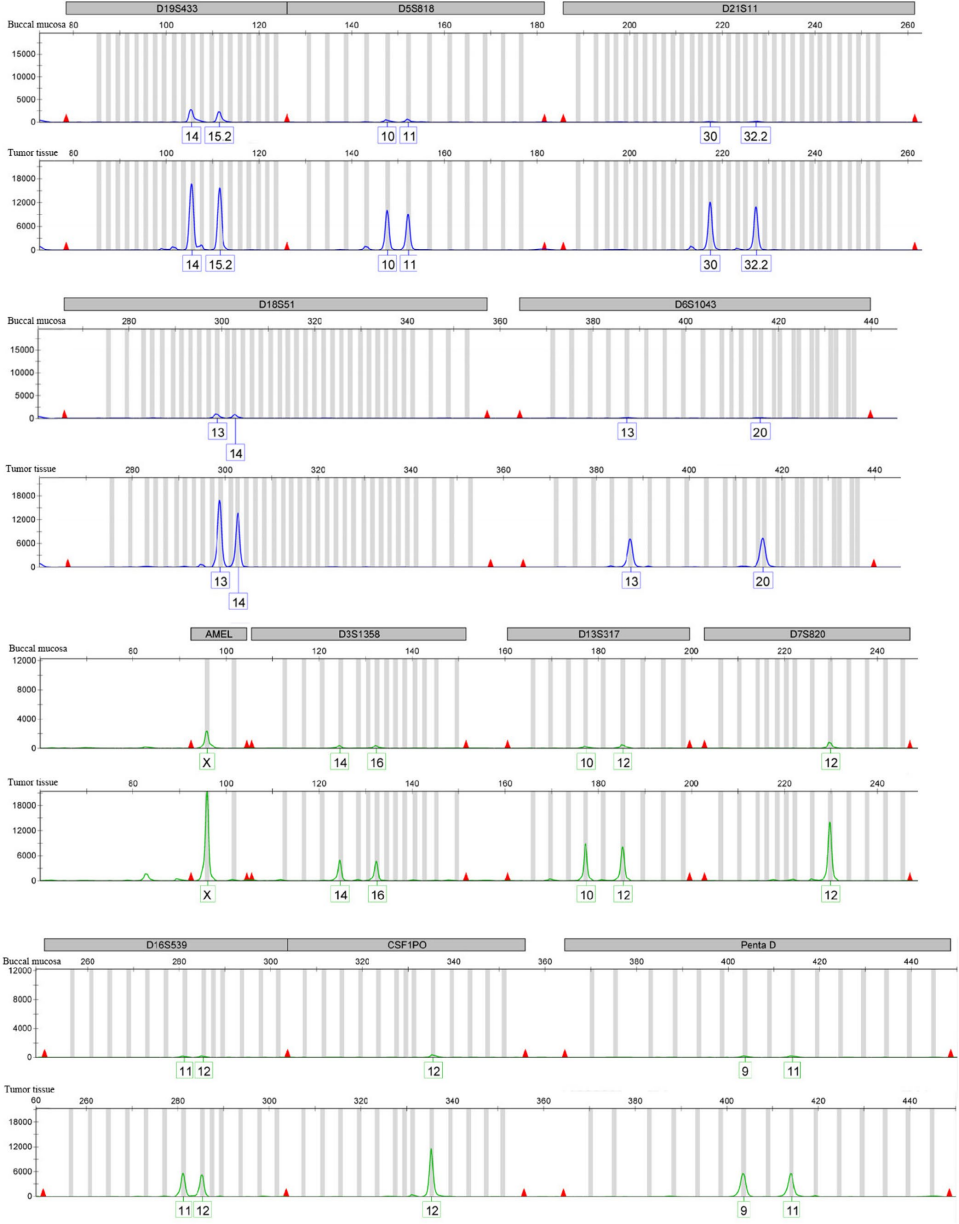
The tumor tissues exhibit identical STR loci profiles to those of the patient's buccal mucosa across all examined loci, including D19S433, D5S818, D21S11, D18S51, D6S1043, D3S1358, D13S317, D7S820, D16S539, CSF1PO, and Penta D.

In February 2024, the patient received 5 cycles of etoposide, methotrexate, actinomycin‐D, alternating with cyclophosphamide and vincristine (EMA‐CO) chemotherapy with subsequent hCG decline (Figure 3). However, STR analysis performed in March 2024 comparing tumor tissue with buccal mucosa DNA confirmed NGCC (Figure 4). The multidisciplinary team (MDT) reviewed the case and found diffuse involvement of the middle and lower uterine segments as well as the entire cervix, without parametrial invasion. Although pulmonary metastases could not be definitively excluded, the lymph node lesions and solid pelvic components had significantly regressed. The MDT concluded that chemotherapy had been effective, with disease currently confined to the uterus and cervix. A total hysterectomy with bilateral salpingo‐oophorectomy was recommended and subsequently performed in April 2024.

Surgical pathology showed chemotherapy‐treated choriocarcinoma in the lower uterine segment and cervical canal, with extensive necrosis and minimal residual trophoblastic cells. A total of 34 lymph nodes were dissected, including 3 from the bilateral common iliac and 31 from the bilateral pelvic regions, all of which were negative for metastases. Postoperative immunohistochemistry results showed: AE1/AE3 (scattered +), GATA‐3 (scattered+), CD146 (scattered+), CK8/18 (scattered+), HSD3B1 (scattered+), HLA‐G (−), hCG (−), Ki‐67 (−), and P63 (−). Final staging was II (T2N0M0), with postoperative hCG declining to 2.99 IU/L (reference range: 0–5 IU/L) within 3 weeks in May 2024.

Given the demonstrated chemosensitivity as evidenced by both pathological response and serial hCG monitoring (Figure 3), the MDT recommended three additional EMA‐CO cycles postoperatively. The patient consented after thorough discussion. At last follow‐up in December 2024, hCG remained normal (3.07 mIU/mL), with no evidence of disease on pelvic MRI (Figure 2B), upper abdominal MRI, or chest CT (Figure 2D). The patient's primary adverse events during the treatment were grade III–IV myelosuppression (marked leukopenia and neutropenia) after chemotherapy, which resolved after secondary prophylaxis; and grade II oral mucositis, which responded to symptomatic treatment. Other reported symptoms included insomnia, postprandial abdominal pain, sore throat, toothache, gingival swelling and tenderness, generalized myalgia, and decreased appetite.

## 3 Discussion

**FIGURE 5:**
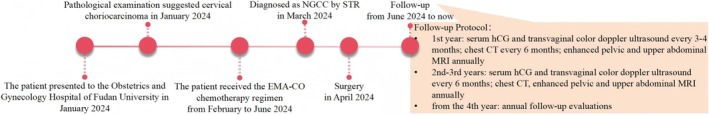
Clinical course and follow‐up protocol of the patient with non‐gestational choriocarcinoma (NGCC). The patient was admitted to the Obstetrics and Gynecology Hospital of Fudan University in January 2024, after pathological examination suggested cervical choriocarcinoma. STR analysis confirmed the diagnosis of NGCC in March 2024. From February through June 2024, she received 6 cycles of EMA‐CO chemotherapy. Since June 2024 she has remained disease‐free on surveillance. Follow‐up schedule: (1) Year 1: Serum hCG and trans‐vaginal color Doppler ultrasound every 3–4 months, chest CT every 6 months, and contrast‐enhanced pelvic plus upper‐abdominal MRI annually; Years 2–3: Serum hCG and ultrasound every 6 months with annual chest CT and MRI; from Year 4 onward: Annual evaluation only.

NGCC is a rare yet highly aggressive malignancy that is frequently misdiagnosed in clinical practice. Early and accurate diagnosis is critical for implementing timely interventions that may prevent disease progression and improve prognosis. We retrieved case reports of NGCC involving the corpus uteri and/or cervix from PubMed published between 1971 and the present. A total of 10 patients were reported [3, 4, 5, 6, 7, 8, 9, 10, 11, 12], with ages from 4 to 65 years. Only one reported case involved primary lesions invading both the corpus uteri and the cervix. All patients underwent surgery and chemotherapy, although the specific regimen was unknown for one patient. Six of the 10 cases were diagnosed using STR (Table 1). Compared with the previously reported cases, ours is the only one describing NGCC with simultaneous uterine and cervical involvement, confirmed by STR, and treated with the EMA‐CO regimen (Figure 5). In summary, the distinctiveness of our case is twofold. Firstly, the lesion was initially detected at a specific anatomical site and definitively diagnosed as NGCC through STR analysis. Second, based on the patient's favorable response to the pre‐operative EMA‐CO regimen, we elected to continue this chemotherapy postoperatively rather than switch to the standard regimen for uterine corpus cancer (carboplatin plus paclitaxel, TC), adhering to the principle of individualized treatment. This case offers valuable insights into the accurate diagnosis of dual‐site NGCC, supports the efficacy of the EMA‐CO regimen, and underscores the current lack of awareness regarding NGCC differential diagnosis, thereby reinforcing the importance of STR in clinical practice.

**TABLE 1:** Case reports of NGCC of the corpus uteri and (or) cervix.

First author, publication year	Age	Tumor location	Surgery	Initial chemotherapy regimen	Alternative chemotherapy regimen	Molecular diagnosis	Outcome
Maestá et al. 2005 [3]	32	Uterine cervix	RH	EMA‐CO	EP‐EMA	STR	DOD 12M
Yildiz et al. 2009 [4]	65	Uterine	TAH; RSO	EMA‐CO	Methotrexate and folinic acid	No	WER 20M
Longo et al. 2011 [5]	54	Uterine cervix	RH	BEP	VIP	No	DOD 7M
Hirata et al. 2012 [6]	58	Uterine and uterine cervix	TAH; BSO	MEA	No	STR	WER 3Y
Wang et al. 2014 [7]	62	Uterine	TAH; BSO	Unknow	Unknow	STR	DOD 12M
Wu et al. 2018 [8]	56	Uterine	RH; BSO; BPLND; PWC	EMA‐CO	No	STR	WER 12M
Sano 2018 [9]	46	Uterine	TAH; BSO	EMA‐CO	EP‐EMA and TP‐TE	STR	DOD 12M
Wang 2019 [10]	63	Uterine	TAH; BSO	EMA‐CO	No	STR	WER 11M
Coutinho et al. 2022 [11]	37	Uterine	TAH; BS	EMA‐CO	No	No	WER
Darmawan et al. 2024 [12]	4	Uterine	RH	Carboplatin; etoposide; bleomycin		No	Recurrence

Abbreviations: BEP, cisplatin + etoposide + bleomycin; BPLND, bilateral pelvic lymph node dissection; BS, bilateral salpingectomy; BSO, bilateral salpingo‐oophorectomy; DOD, dead of disease; EMA‐CO, etoposide + methotrexate + actinomycin D + cyclophosphamide + vincristine; EP‐EMA, etoposide + cisplatin + methotrexate + actinomycin D; M, months; MEA, methotrexate + etoposide + actinomycin D; PWC, pelvic wash cytology; RH, radical hysterectomy; RSO, right‐sided salpingo‐oophorectomy; STR, short tandem repeat; TAH, total abdominal hysterectomy; TP‐TE, paclitaxel + cisplatin + paclitaxel + etoposide; VIP, cisplatin + vinblastine + ifosfamide; WER, without evidence of recurrence; Y, year.

NGCC and GCC differ significantly in terms of susceptible population, reproductive history, treatment regimen selection, and prognosis. GCC primarily affects women aged over 25, with incidence peaking after age 39. NGCC demonstrates a bimodal distribution, occurring in teenagers, young reproductive‐age women, postmenopausal women, and occasionally males. Regarding diagnosis, GCC tumors show STR allelic patterns discordant with the patient's genome but partially matching the sexual partner's, while NGCC tumors are genetically identical to the patient. GCC responds well to EMA‐CO chemotherapy as first‐line treatment, whereas NGCC requires radical surgical resection as primary therapy. In terms of prognosis, GCC has 82%–100% overall survival rates, contrasting sharply with NGCC's 23.5% mortality and 17.6% recurrence rates. Additionally, GCC is pregnancy‐associated, while NGCC develops independently of gestation [13, 14, 15, 16, 17]. The diagnostic challenge between NGCC and GCC is due to the overlapping histopathological features, nonspecific clinical presentations, and lack of definitive diagnostic criteria. Some studies propose using the interval between first pregnancy and cancer onset as a diagnostic indicator [2], though molecular testing such as polymorphic microsatellite and STR analyses offer greater reliability [18].

In clinical practice, STR serves as an effective and feasible molecular diagnostic tool. It can reliably differentiate gestational from non‐gestational choriocarcinoma, thereby complementing the FIGO score and optimizing clinical management [19]. Furthermore, STR is valuable in the differential diagnosis of partial hydatidiform moles, hydropic abortions, and miscarriages from other causes [20]. Beyond these applications, STR genotyping can also identify disease tissue origin. For instance, one study successfully utilized STR markers to detect genomic alterations and precisely delineate potential loss of heterozygosity (LOH) loci in a mixed germ cell tumor [21].

## 4 Conclusion

Our case is distinctive in several key aspects. The patient was initially misdiagnosed with GCC based on biopsy findings, leading to the initiation of EMA‐CO chemotherapy. Subsequent STR analysis and postoperative histopathology, however, confirmed NGCC. This reclassification prompted surgical intervention, after which chemotherapy was continued. This individualized treatment strategy, developed through MDT discussion and shared decision‐making with the patient, achieved complete remission, which has been sustained over 6 months of follow‐up.

This experience underscores the essential role of molecular diagnostics such as STR analysis in differentiating NGCC from GCC, particularly given their frequently overlapping clinical and pathological presentations. Accurate genotyping enabled the design of a personalized treatment plan based on the primary tumor site, pathological features, and the early treatment response. Collectively, this case provides valuable insights for establishing a personalized therapeutic framework for NGCC, and highlights the critical synergy between molecular diagnostics and tailored clinical management in this rare malignancy.

## Author Contributions

**Jiahui Ma:** formal analysis, investigation, visualization, writing – original draft. **Fenghua Ma:** data curation, investigation, writing – review and editing. **Tingting Chen:** data curation, investigation, writing – review and editing. **Xin Lu:** funding acquisition, resources, supervision, writing – review and editing. **Yan Du:** funding acquisition, project administration, supervision, writing – original draft, writing – review and editing. **Xiaoni Yue:** conceptualization, investigation, supervision, validation, writing – review and editing.

## Ethics Statement

This case report has been approved by the Ethics Committee of the Obstetrics and Gynecology Hospital of Fudan University (Approval No.: FCKIRB‐2023‐135).

## Consent

We have obtained informed consent to publish this report from the patient.

## Conflicts of Interest

The authors declare no conflicts of interest.

## Data Availability

The data that support the findings of this study are available on request from the corresponding author. The data are not publicly available due to privacy or ethical restrictions.
